# Quantitative Phosphoproteomics Reveals Cell Alignment and Mitochondrial Length Change under Cyclic Stretching in Lung Cells

**DOI:** 10.3390/ijms21114074

**Published:** 2020-06-07

**Authors:** Wei-Hsuan Wang, Chia-Lang Hsu, Hsuan-Cheng Huang, Hsueh-Fen Juan

**Affiliations:** 1 Genome and Systems Biology Degree Program, Academia Sinica and National Taiwan University, Taipei 10617, Taiwan; weihsuan15@gmail.com; 2 Department of Life Science, National Taiwan University, Taipei 10617, Taiwan; chialanghsu@ntuh.gov.tw; 3 Department of Medical Research, National Taiwan University Hospital, Taipei 10002, Taiwan; 4 Institute of Biomedical Informatics, National Yang-Ming University, Taipei 11230, Taiwan; 5 Graduate Institute of Biomedical Electronics and Bioinformatics, National Taiwan University, Taipei 10617, Taiwan

**Keywords:** quantitative phosphoproteomics, cyclic stretching, cytoskeleton reorganization, mitochondrial length

## Abstract

Lung cancer is a leading cause of death. Most previous studies have been based on traditional cell-culturing methods. However, lung cells are periodically subjected to mechanical forces during breathing. Understanding the mechanisms underlying the cyclic stretching induced in lung cells may be important for lung cancer therapy. Here, we applied cyclic stretching to stimulate the continual contraction that is present under physiological conditions in lung cells. We first uncovered the stretching-induced phosphoproteome in lung cancer cell line A549 and fibroblast cell line IMR-90. We identified 2048 and 2604 phosphosites corresponding to 837 and 1008 phosphoproteins in A549 and IMR-90, respectively. Furthermore, we combined our phosphoproteomics and public gene expression data to identify the biological functions in response to cyclic stretching. Interestingly, cytoskeletal and mitochondrial reorganization were enriched. We further used cell imaging analysis to validate the profiling results and found that this physical force changed cell alignment and mitochondrial length. This study not only reveals the molecular mechanism of cyclic stretching but also provides evidence that cell stretching causes cellular rearrangement and mitochondrial length change.

## 1. Introduction

Cyclic stretching is a process that can be investigated in adherent cells seeded on a flexible surface and then stretched periodically. This procedure creates a dynamic environment that simulates that of continually contracting organs, such as the lungs, heart, and muscle tissue. This stretching force is important for organ development. The change in the volume of the breathing lung is necessary for the lung mechanism, function, and growth [1]. Previous studies suggest that many cells and tissues respond to cyclic stretching [2,3,4]. For example, the force-driven enrichment of the mechanosensory complex of emerin results from the recruitment of myosin 2A and local actin. It affects the organization of chromatin and controls lineage commitment in stem cells [4]. The transmembrane protein Piezo1, in dog epithelial cells, activates mitosis after stretching through the stimulation of cyclin B [5].

Phosphorylation is an essential aspect of cells. For example, somatic mutations of epidermal growth factor receptors (EGFRs), a receptor tyrosine kinase on the plasma membrane, leads to autophosphorylation and constitutive activation, resulting in incoercible cell division in cancers [6]. The characterization of the protein phosphorylation status underlying stimulus-induced signaling changes may provide important insights into the regulation of physiological events in cells. Recent advances in quantitative phosphoproteomic profiling allow researchers to study the aberrant regulation of signaling pathways [7,8,9,10].

Several studies have shown that stretching activates signaling pathways. For instance, stretched muscle cells activate the Nox4 signaling pathway [11] and enhance nuclear protein import [12]. The proliferation of mesenchymal stem cells is induced by the stretch-driven focal adhesion kinase (FAK)- extracellular regulated protein kinases (ERK)1/2 signaling pathway [13]. Monolayer permeability can be modulated by the stretching-induced mitogen-activated protein kinase (MAPK) signaling pathway in rat alveolar epithelial cells, which is mainly because MAPK signaling transduction activates tight junction-related protein expression [14]. Other research suggests that cyclic stretching induces ROS-activated protein kinase C (PKC) activity, resulting in the FAK-driven downstream signaling pathway [15]. In addition, in cardiac myocytes, stretching influences the activity of RhoA signaling and the gastrin-mediated signaling pathway through their transcriptomic profiles [16].

In the present study, we first applied the phosphoproteomic technique to profile the stretching-induced phosphoproteome in A549 and IMR-90 cells. Furthermore, we analyzed differential phosphoproteomic statuses using bioinformatics tools and statistical methods, thus revealing stretching-induced biological functions. The study shows that the stretching state is different from the static state, not only for cancer cells, but also for normal fibroblasts.

## 2. Results

**Figure 1:**
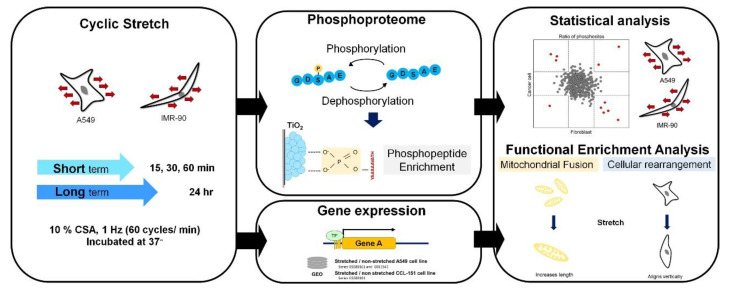
Experimental procedure and workflow of cyclic stretching-induced phosphoproteomics analysis. In this study, we applied short-term (15, 30, and 60 min) and long-term (24 h) stretching to two cell lines. Collected proteins were digested and processed, then enriched for phosphopeptides using TiO_2_. Phosphopeptides were analyzed by mass spectrometry. Phosphoproteome data were quantified and identified using MaxQuant software. We performed the functional enrichment of stretching-induced genomic and phosphoproteomic differential expressed genes/proteins.

The experimental workflow is illustrated in Figure 1. In this study, we applied different time scales of cyclic stretching to gain insight into the stretching-induced phosphoproteome in lung cancer cell line A549 and lung fibroblast line IMR-90. Due to the relatively low proportion of phosphopeptides compared to peptides, phosphopeptides were enriched by hydroxy acid-modified metal oxide chromatography (HAMMOC) [17]. After nanoscale liquid chromatography–tandem mass (LC–MS/MS) spectrometry analysis, we performed the statistical analysis and functional assay. The results showed that several stretching-induced biological processes have significant differences. Additionally, stretching-induced mitochondrial fusion and cellular rearrangement are further validated by cellular experiments based on imaging.

### 2.1. Cell Surface Area (CSA) Determination

Larger CSA changes may cause apoptosis [15,18]. To determine the stable stretching state, we first confirmed the stability of the CSA to ensure that the stretching would not affect cell survival. Several cyclic stretching-related studies are summarized in Table S1. CSA changes of 10–20% correspond to 70–80% changes in total lung capacity, according to morphometric analysis [19]. Additionally, 20% and 30% changes in CSA resulted in apoptosis in human and rat cells [20,21]. According to previous reports, 10% CSA and a uniaxial stretching direction were commonly applied in cyclic stretched lung cells [22,23,24,25,26,27,28]. Therefore, 10% CSA change and a uniaxial stretching direction are used for the cyclic stretching experiment.

### 2.2. Quantitative Phosphoproteome of Lung Cells in Response to Cyclic Stretching

**Figure 2:**
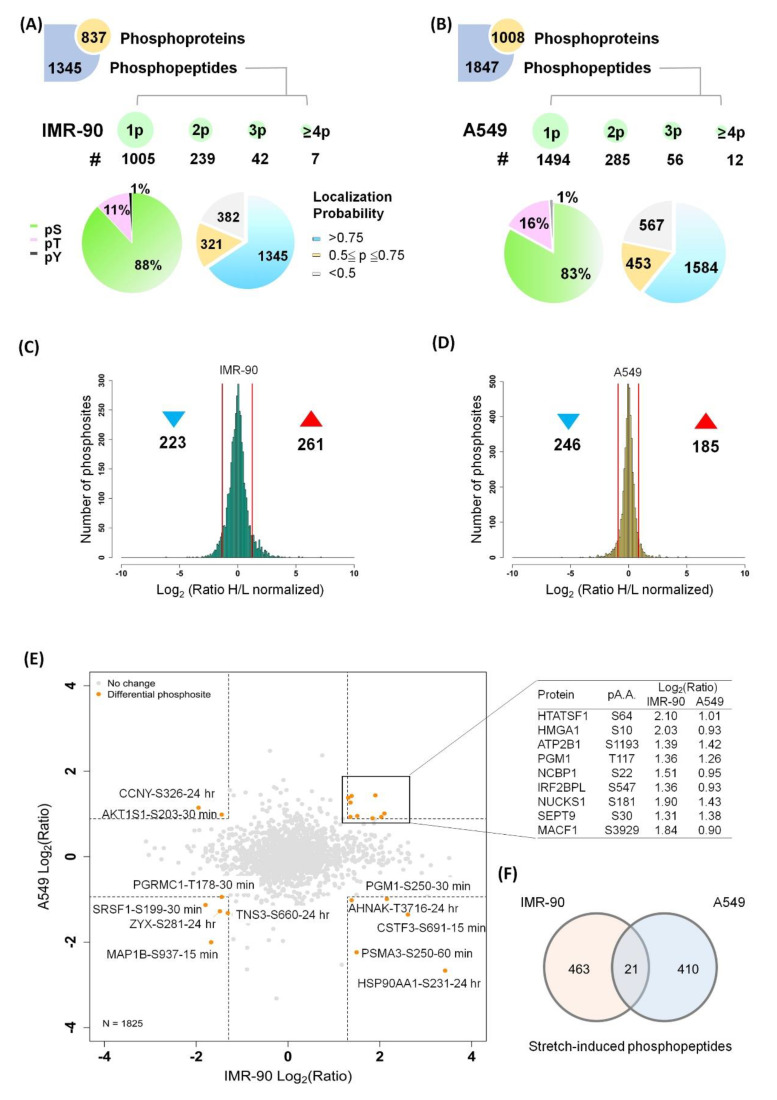
Quantitative phosphoproteome profiling of stretched A549 and IMR-90 cells. (**A**,**B**) The quantitation and identification of the phosphoproteome of IMR-90 and A549 lung cells in response to cyclic stretching. In IMR-90, 1345 phosphopeptides, corresponding to 837 phosphoproteins were identified in. One phospho-group identified on one peptide is represented by 1p, 2p represents two phospho-groups identified on one peptide, etc. Phosphorylated residues of serine, threonine, and tyrosine are indicated as pS, pT, and pY. Localization probability represents the reliability of the phosphorylated position, while phosphosites having a probability of more than 0.75 represent a higher confidence. (**C**,**D**) Histogram of log_2_-transformed normalized phosphosites in IMR-90 (**C**) and A549 (**D**) cells. Red lines indicate the threshold of differential phosphosites (mean ± 1.5 SD). Symbol ▲ and the number below it show the up-regulated phosphosites induced by cyclic stretching. Symbol ▼ and the number below it show the down-regulated phosphosites induced by cyclic stretching. (**E**) Scatter plot illustrating the overlapping phosphosites identified in the two cell lines. (**F**) Venn diagram of significantly regulated phosphopeptides that are conserved in cyclic stretching.

Cyclic stretching resulted in a multi-biological function change [29,30,31,32]. A protein phosphorylation cascade is typical in regulating cellular functions. Therefore, we applied a phosphoproteome technique to gain insight into the stretching-induced global phosphoproteome. The two cell lines were stretched for 15, 30, 60 min or 24 h at 1 Hz (60 cycles/min) with a 10% CSA change. We identified 2048 phosphosites on 1345 unique phosphopeptides in IMR-90 and 2604 phosphosites on 1847 unique phosphopeptides in A549 (Figure 2A,B). Most peptides were singly or doubly phosphorylated, yielding Ser:Thr:Tyr phosphorylation ratios of 88:11:1 and 83:16:1 in IMR-90 and A549, respectively.

To ensure the reliability of downstream analyses, we retained only the phosphosites with a localization probability of >0.75. The differential regulation of 484 (223 down- and 261 up-regulated) and 431 (246 down- and 185 up-regulated) phosphosites was demonstrated in IMR-90 and A549, respectively (Figure 2C,D; Tables S2 and S3). A total of 1825 phosphosites were identified in the two cell lines in the stretching time series experiment. Those phosphosites that showed the same tendency can be considered mechanically stretch-sensitive (Figure 2E). Mass spectra data were deposited at ProteomeXChange (http://www.proteomexchange.org/), project accession number PXD313200. In addition, we also overlapped regulated phosphopeptides in the two cell lines (Figure 2F) There are 21 phosphopeptides that responded to cyclic stretching in the two cell lines.

### 2.3. Functional Enrichment of Stretching-Regulated Phosphoproteins

**Figure 3:**
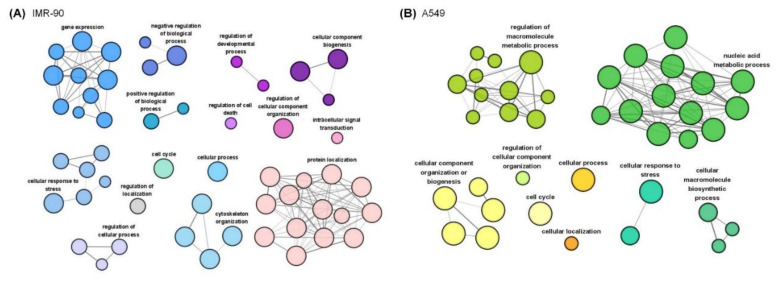
Network analysis of stretching-induced phosphorylation events. Phosphosites with a 1.5-fold change were analyzed with ClueGo (**A**). Network analysis of stretching-regulated phosphoproteins in IMR-90. (**B**). Network analysis of stretching-regulated phosphoproteins in A549.

Phosphoproteins with a 1.5-fold change were analyzed with network analysis using ClueGo [33] in two cell lines (Figure 3A,B). We found that gene expression, the negative regulation of biological processes, and intracellular signal transduction functions are unique in IMR-90 cells. The regulation of the macromolecule metabolic process and nucleic acid metabolic process was specifically enriched in A549 cells. Interestingly, two cells share similar biological functions after cyclic stretching, such as the cellular response to stress, cellular component organization, and cellular processes.

**Figure 4:**
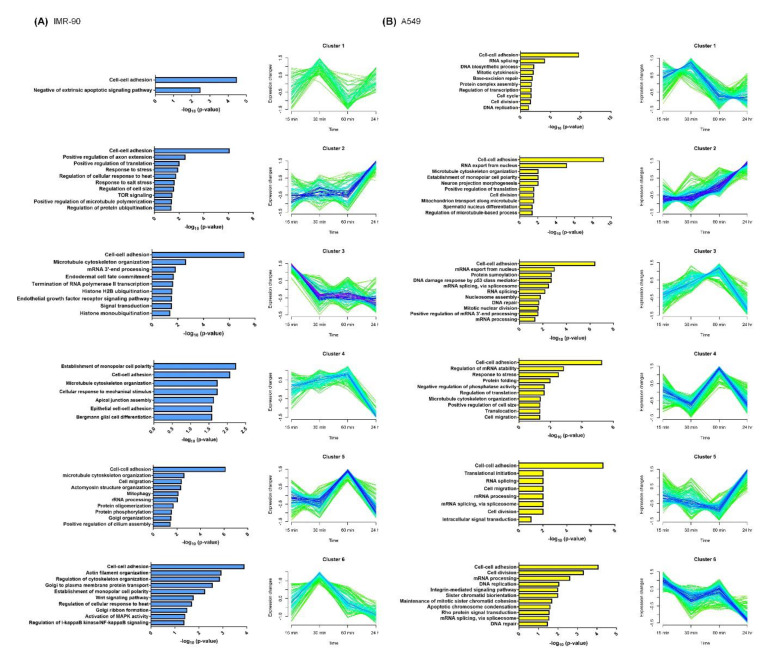
Fuzzy c-means clustering and gene ontology analysis in IMR-90 cells (**A**) and A549 cells (**B**). All phosphosites were classified into six clusters using the bioconductor R package named “Mfuzz”. Blue represents the phosphoproteins that showed significant changes and green means phosphoproteins with no obvious change. Phosphoproteins enriched in different clusters were further analyzed with gene ontology using the Database for Annotation, Visualization and Integrated Discovery (DAVID).

We further classify these identified phosphoproteins into six clusters by Mfuzz [34], based on a time series phosphorylated log_2_-transformed ratio (Figure 4) and analyzed the biological functional enrichment in all clusters using the Database for Annotation, Visualization and Integrated Discovery (DAVID) [35] in the two cell lines. Phosphoproteins participation in cell–cell adhesion is enriched in all clusters in the two cell lines. Many cytoskeleton-related functions are found in the two cell lines, such as the establishment of monopolar cell polarity (cluster 4), microtubule cytoskeleton organization (cluster 5), and actin filament organization (cluster 6) in IMR-90 cells, and microtubule cytoskeleton organization (cluster 2), the regulation of cell size (cluster 4), and cell migration (cluster 6) in A549, respectively.

### 2.4. Functional Enrichment of Stretching-Regulated Gene Expression

According to our phosphoproteomics data, cellular component organization and gene expression-related functions are enriched after continuous stretching. Transcription activation is one of the common downstream results of signaling pathways [36,37]. For instance, transcription factor YAP1 regulates cellular adhesion by activating adhesion-related gene expression [38]. The modification of YAP1-S127 is necessary for its delivery to the nucleus [39]. This further confirms that gene expression-related functions were enriched after continuous stretching in our phosphoproteomics data. Thus, we added public gene expression data to reveal the downstream gene expression profiles that were induced by stretching.

Santos et al. generated gene expression signatures of A549 treated with TNFα, lipopolysaccharide, and cyclic stretching (GEO accession number: GSE15411) [40]. Here, we used the GSE15411 dataset for the cells under physical stretching as a control group. The other dataset provides gene expression data for A549 cells and the CCL-151 lung fibroblast cell line (GEO accession number: GSE80161). Since there is no GEO data resource of a stretched IMR-90 cell line, we applied the other lung fibroblast CCL-151 cell line instead. We identified 1634 and 503 significantly regulated genes out of 20,967 and 20,358 genes in the A549 and CCL-151 cell lines, respectively (Figure S1A,B). We found 130 and 108 over-represented Gene Ontology (GO) terms with a normalized *p*-value of <0.05 in IMR-90 and A549 cells, which are illustrated in Figure S2C.

Protein maturation, phosphorylation, and organelle organization were the unique functions in A549, whereas the cellular amide metabolic process, mitochondrion organization, and the negative regulation of phosphorylation were enriched in CCL-151. Several enriched functions in the gene expression datasets corresponded to our phosphoproteome data, such as cell communication, cytoskeleton organization, and organelle transportation. These findings suggest that the stretching force induced similar responses at the levels of gene expression and protein modification.

### 2.5. Uniaxial Cyclic Stretching Resulted in Cell Rearrangement

**Figure 5:**
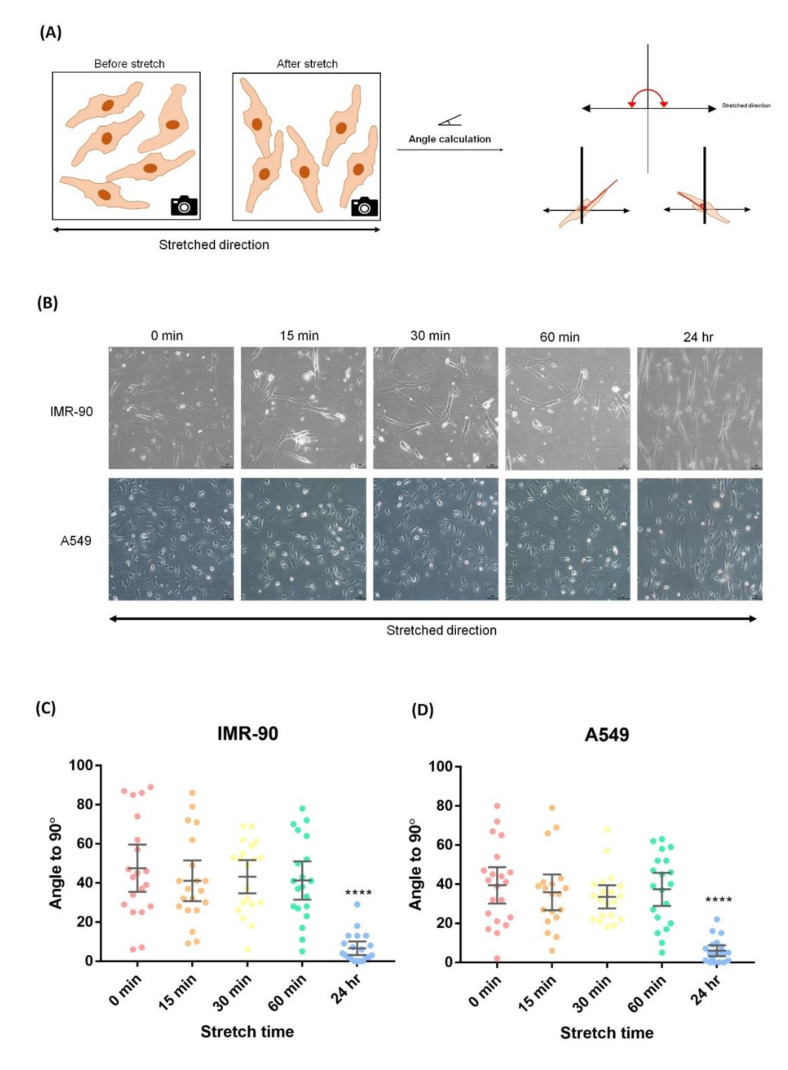
Image-based quantification of cyclic stretching-induced cellular rearrangement. (**A**) Schematic of the angle quantification process. The angle was between 0° and 180° with respect to the axis of stretch. (**B**) Cells subjected to different periods (0, 15, 30, 60 min and 24 h) of stretching. The arrow indicates the direction of stretching. (**C**,**D**). Scatter plot of the orientation of IMR-90 and A549 cells after different periods of cyclic stretching. The angle was measured between 0° and 180° with respect to the axis of stretching then 90 ° was subtracted and the absolute value was taken, which indicated the angle to the vertical. Twenty cells from each time period were selected for the measurement. The angle of the two cell lines were distributed evenly before stretching and moved to be vertical to the stretching direction after 24 h of stretching. An unpaired two-tailed Student’s *t*-test was used, where a *p*-value < 0.05 was considered as a significant change.

Our functionally enriched data showed that cyclic stretching induced cytoskeleton reorganization in both IMR-90 and A549 cells. Cytoskeleton reorganization is related to cellular movement. Therefore, we further observed the cell morphology and measured the cellular growth angle after cell stretching using ImageJ software (Figure 5A). The cell morphologies in Figure 5B showed that uniaxial cyclic stretching resulted in cell rearrangement. Most of the cells treated with stretching for 24 h arranged themselves vertically with respect to the direction of stretching (Figure 5C,D). This phenomenon indicates that cellular reorientation is a common effect caused by uniaxial stretching.

### 2.6. Cyclic Stretching Enhanced Mitochondrial Length

The results of the functional enrichment of both gene expression and the phosphoproteome showed that the stretching altered mitochondrial organization and mitochondrial transportation. Mitochondria generate ATP, which is essential for cell survival. Changes in mitochondrial morphology help to maintain their health, and this process includes mitochondrial fission (fragmentation) and fusion [41,42].

**Figure 6:**
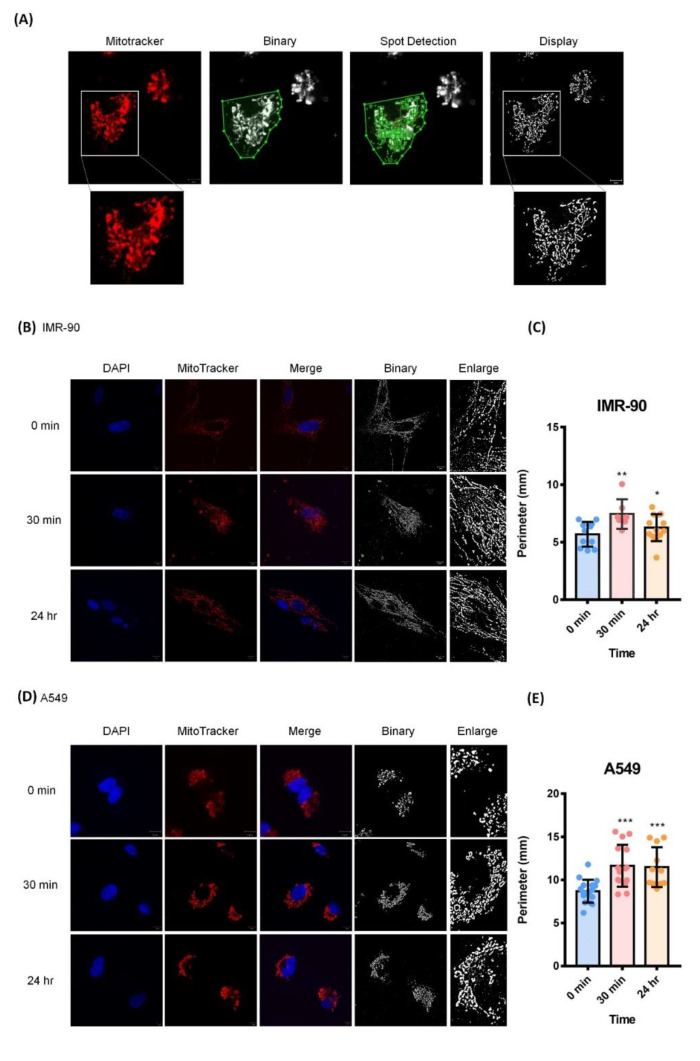
Mitochondrial lengths were enhanced by cyclic stretching. (**A**) A display of MitoTracker-stained images, binary images, the spot detection procedure, and the images after calculation. (**B**) The immunofluorescence images of stretched IMR-90. Mitochondria were stained with MitoTracker during stretching. Cells were fixed with 3.7% paraformaldehyde after stretching for different durations. The blue and red panels indicate cellular nuclei and mitochondria, respectively. (**C**) Statistical tests of mitochondrial perimeters, comparing the static and stretched groups in IMR-90. *, **, and *** mean a *p*-value < 0.05, < 0.01, and < 0.005, respectively. (**D**) The immunofluorescence image of stretched A549. The blue and red panels indicate cellular nuclei and mitochondria, respectively. (**E**) Statistical tests of mitochondrial perimeters, comparing the static and stretched groups in A549. *, **, and *** mean a *p*-value < 0.05, < 0.01, and < 0.005, respectively.

Here, we further applied immunocytochemistry (ICC) to investigate whether mitochondrial fission or fusion was induced by mechanical stretching. We analyzed ICC images via bio-image analysis using the software Icy [43]. Icy software can transform immunostaining images into binary panels for further calculation. Mitotracker-stained images were processed by several steps and quantified. The image can be output after the statistical calculation (Figure 6A). The morphologies of mitochondria after different stretching time series are shown in Figure 6B,D. We observed that the mitochondrial lengths were enhanced after cyclic stretching in both A549 and IMR-90 cells. We further measured the mitochondrial perimeters and performed statistical tests [44,45] (Figure 6C,E). The results indicated that mitochondria became significantly longer in both cell lines after 30 min and 24 h of stretching.

### 2.7. Global View of the Phosphorylation Events Induced by Cyclic Stretching

**Figure 7:**
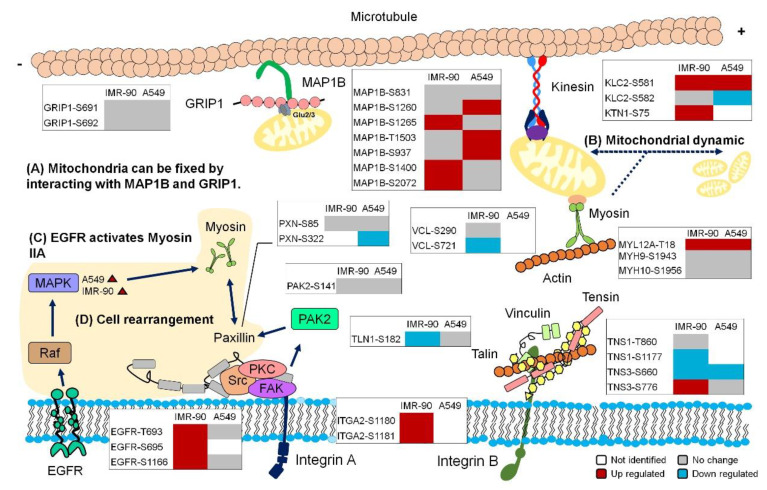
Cyclic stretching induces cellular rearrangement and enhances mitochondrial length. Graphical summary of this study. Several of the phosphosites of essential proteins that were significantly regulated under cyclic stretching may participate in specific biological functions. (**A**) Mitochondrial movement could be slowed down by the interaction between the microtubule-associated protein 1B (MAP1B) and glutamate receptor-interacting protein 1 (GRIP1). (**B**) Myosin participates in mitochondrial fragmentation. (**C**) Activation of Epidermal growth factor receptor (EGFR) signaling enhances the activity of myosin IIA. (**D**) Several focal adhesion-related proteins were activated by cyclic stretching in both cell lines. The change in focal adhesion activity resulted in cellular rearrangement.

From our phosphoproteomics data, the relationships between cyclic stretching, mitochondrial length, and cellular rearrangement are shown in Figure 7. Mitochondrial motility could be assessed using the binding of GRIP1-associated protein 1 (GRIPAP1) and kinesin [46]. Although mitochondria tend to be fixed somewhere in the cell, the interaction between microtubule-associated protein 1B (MAP1B) and GRIPAP1 aid their anchoring on microtubules [47]. The other trajectory for mitochondrial motility is climbing along actin through interactions with myosin [48,49]. The contribution of these two cytoskeletal components to mtDNA maintenance has been reported [50]. Furthermore, myosin II plays an important role in mitochondrial fission. Disrupting the expression of myosin IIA and IIB causes a significant decrease in mitochondrion-associated Drp1 [51]. The upstream regulation of fission includes integrin receptor-induced focal adhesion and cytoskeletal rearrangement. Talin serves as a mechanosensor by directly interacting with integrin [52]. Talin and tensin are cytoskeletal components known for regulating focal adhesion in response to mechanical stretching [53]. The other components of this pathway include FAK, PKC, and Src. The epidermal growth factor receptor (EGFR) acts as another mechanosensory receptor [54,55]; it activates myosin II through the MAPK signaling pathway. The global activation of these cytoskeletal components and focal adhesion may induce cross-talk with the EGFR-induced MAPK pathway, resulting in a change in focal adhesion, thus inducing cellular rearrangement [56,57,58].

## 3. Discussion

Several cyclic stretching-induced effects have been discussed previously, such as cell proliferation, the disruption of cell–cell communication, and microtubule reorganization [24,59,60,61]. These biological functions are activated by different signaling pathways [13,60,62,63,64]. However, the cyclic stretching-induced global phosphoproteome remains lacking.

In the present study, we have provided a global view of the stretching-induced phosphoproteome by stretching lung cancer cell line A549 and fibroblast cell line IMR-90 for short periods (15, 30, or 60 min) and a longer period (24 h). Some phosphosites showed sensitivity to stretching in both cell lines, such as SEPT9 Ser30 and Zyxin Ser281. The phosphorylation of SEPT9 Ser30 promotes cell adhesion in HeLa cells [65]. Previous research has shown that stretching disrupts tight junctions and adhesion [61,66]. SEPT9 Ser30 was up-regulated in both the IMR-90 and the A549 cells. Therefore, we suppose that this phosphorylation contributes to the response of the cells to the stretching. Similarly, phosphorylated Zyxin Ser281 interacts with CDK8, thus activating YAP-mediated mitosis [67]. This phosphosite showed down-regulation in the two cell lines. Cells delay proliferation when they face external stress [68]. The other phosphosites showed different degrees of modification. The AKT1S1S Ser203 phosphosite has been reported to activate the mTORC signaling pathway, and thus enhance oncogenic growth [69]. This phosphosite was up-regulated in the A549 cancer cells, but was down-regulated in the IMR-90 fibroblasts.

In the functional network analysis, we found that cytoskeleton organization and cellular component organization are enriched in both A549 and IMR-90 cells. To get an insight into the biological functions induced by cyclic stretching, we applied clustering analysis and found the cell–cell adhesion function showed the significant enrichment in six clusters in both cell lines. Focal adhesion proteins regulated by mechanical stretching through a mechano-sensor were reported [58]. This stretching force stimulated adhesion molecule expression in the extracellular matrix thus enhanced cell–cell communications [61]. Several cytoskeletons were involved in this function, which may be related to cellular mobility or rearrangement; therefore, we further investigated whether cellular movement occurs after cyclic stretching. Indeed, we observed cellular rearrangement after cyclic stretching. This phenomenon was also found in vascular endothelial and fibroblast cells [26,28].

Cellular rearrangement is induced by the mechanical stretching that frequently occurs in various cell types, such as muscle cells and osteoblasts [2,59]. Interestingly, the phenomenon was also observed in A549 and IMR-90 cell lines, which were used in our study. Cellular reorientation is a common response to cyclic stretching, and consistent with that, we identified several significantly altered phosphosites on proteins involved in filament polymerization and synthesis, including actin filaments, myosins, and microtubules. We suggest that these phosphoproteins may be related to cellular rearrangement induced by cyclic stretching.

Moreover, we found that mitochondria and Golgi transportation and their biosynthesis are the result of functional enrichment. Combined with the stretching-induced gene expression and functional annotation, we found that the function related to mitochondrial organization was regulated by cyclic stretching. In recent years, mitochondrial dynamics, such as fusion and fission, has become popular subjects and are involved in the maintenance of mitochondrial health [70,71]. Previous research found that mitochondria elongated their length after cyclic stretching in cardiac cells [72]. Mitochondrial dynamics were also reported as mechano-sensors, which tend to undergo fusion in response to stretching forces in smooth muscle cells [73]. Our data showed that the 30 min and 24 h stretching could elongate mitochondrial length, which may be related to mitochondrial dynamics in both IMR-90 and A549 cells. Our results are consistent with previous studies.

In the present study, our phosphoproteome data reveal the cell type-specific and common phosphosites that respond to cyclic stretching. In addition, we proved that cell realignment is a strategy for cells to resist continual stretching and showed that mitochondrial length was enhanced by cell stretching. We suggest that cyclic stretching-induced phospho-level changes may provide a new perspective on dynamic cell stretching.

## 4. Materials and Methods

### 4.1. Cell Cultures

Human A549 lung carcinoma cells and IMR-90 lung fibroblasts were obtained from the American Type Culture Collection (ATCC, Manassas, VA, USA). These two cell lines were cultured in Dulbecco’s modified Eagle medium (DMEM, Gibco Laboratories, Grand Island, NY, USA) supplemented with 10% fetal bovine serum (FBS, Biological Industries, Kibbutz Beit Haemek, Israel). All cells were grown at 37 °C with 5% CO_2_.

### 4.2. Selection of Cyclic Stretching Conditions

For all stretching-related experiments, the two cell lines were plated in poly-L-lysine-coated flexible chambers at a density of 5.0 × 10^4^ cells/cm^2^ for 24 h. The cells were then subjected to cyclic stretching with a 10% cell surface area (CSA) change, at 1 Hz, using the ATMS Boxer Cyclic Stretching Culture System (Genemessenger, Kaohsiung, Taiwan). Cells were cultured under the same conditions as the static controls, at 37 °C with 5% CO_2_, during stretching.

### 4.3. Cellular Orientation Measurement

Cellular rearrangement was quantified using the angle tool in ImageJ version 1.52a (National Institutes of Health [NIH], Bethesda, MD, USA). We measured the cellular angle perpendicular to the direction of stretching before and after stretching.

### 4.4. Protein Extraction

Cells were washed twice with PBS and extracted using 12 mM sodium deoxycholate (Sigma-Aldrich, St. Louis, MO, USA), 12 mM sodium lauroyl sarcosine, 50 mM triethylammonium bicarbonate (Sigma-Aldrich, St. Louis, MO, USA), protease inhibitor cocktail (BioShop, Burlington, ON, Canada), and phosphatase inhibitor cocktail (Sigma-Aldrich, St. Louis, MO, USA). The cells were homogenized on ice using a homogenizer (LABSONIC M ultrasonic homogenizer, Satorius AG, Goettingen, Germany) with 60% amplitude and a 0.6 s cycle duration (i.e., operated for 0.6 s and rested for 0.4 s) for 1 min. The supernatant containing the protein extract was collected by centrifugation at 16,000× *g* for 20 min at 4 °C. The protein concentration was measured using a Pierce BCA Protein Assay kit (Thermo Fisher Scientific, Waltham, MA, USA) according to the manufacturer’s protocols.

### 4.5. Phosphoproteome Experiments

All phosphoproteome experiments were performed according to our previous studies [10,74]. Five hundred micrograms of protein lysate were used for the phosphoproteome experiments. Protein lysate was first reduced by 0.5 M dithiothreitol and 1 M iodoacetamide, alkylated by Lys-C, and then digested by trypsin. One hundred micrograms of digested peptides were labeled with either 4% (*v*/*v*) formaldehyde-H2 (Sigma-Aldrich, St. Louis, MO, USA) or 4% (*v*/*v*) formaldehyde-D2 (Sigma-Aldrich, St. Louis, MO, USA). In this experiment, the control static group was labeled with 4% (*v*/*v*) formaldehyde-H2 and the stretching groups were labeled with 4% (*v*/*v*) formaldehyde-D2. Then the peptides of the control group and each stretching group were mixed. The phosphopeptides were enriched with hydroxy acid-modified metal oxide chromatography (HAMMOC) [17,75,76].

### 4.6. Nano LC-MS/MS Analysis

Nano LC–MS/MS analysis was performed on a nanoACQUITY UPLC system (Waters, Milford, MA, USA) connected to an LTQ-Orbitrap XL hybrid mass spectrometer (Thermo Electron, Bremen, Germany) equipped with a nanospray interface (Proxeon, Odense, Denmark). Peptides were loaded onto a 2 cm × 180 µm capillary trap column and then separated in a 75 µm × 25 cm nanoACQUITY 1.7 µm BEH C18 column (Waters, Milfrad, MA, USA) at a flow rate of 300 nL/min. Mobile phase A consisted of 0.1% formic acid (Wako, VA, USA) and B consisted of 0.1% formic acid and 80% acetonitrile (ACN) (Thermo Fisher Scientific, Waltham, MA, USA) A linear gradient of 10% to 40% B in 90 min and 40% to 85% B in 10 min was employed throughout this study. Mass spectra from survey full scans were acquired on the Orbitrap (m/z 350–1500). The resolution was 60,000 at m/z 400 and the automatic gain control (AGC) was set to 1 × 10^6^ ions. The top ten most intense precursor ions were selected from the MS scan for subsequent collision-induced dissociation MS/MS scans by ion trap (AGC target at 7000).

### 4.7. Phosphoproteomics Data Processing and Analysis

Raw mass spectral information was processed for peak detection and quantification using MaxQuant software version 1.5.3.0 (Martinsried, Germany) [77]. Peptide identification was performed using the Andromeda search engine against the Swiss-PROT human database, canonical version (published 10 May 2017). Search criteria used in this study were trypsin specificity, fixed modification of carbamidomethyl (C), and variable modifications of oxidation (M) and phosphorylation (STY). Two or more missed cleavages were allowed. Peptide lengths of at least six amino acids were required. The precursor mass tolerance was 3 ppm, and the fragment ion tolerance was 0.5 Da. By using the decoy database strategy, peptide identification was accepted based on the posterior error probability with a false discovery rate of 1%. Precursor intensities of previously identified peptides were further searched and recalculated using the “match between runs” option in MaxQuant. For multiple phosphopeptides, the localization probability of all putative phosphosites was determined using the MaxQuant PTM score algorithm. For single peptides with multiple hits, all matched proteins were counted separately. All the spectra and accompanying information were uploaded to the ProteomeXChange database (http://www.proteomexchange.org/) with the project accession PXD012010.

### 4.8. Functional Enrichment Analysis of Differential Phosphoproteins

The significance of the phosphosites was defined using the parameter of normalized H/L ratio, which was greater than or less than 1.5 times the standard deviation (SD) [77]. To annotate the functional profile, we performed Gene Ontology (GO) analysis using Cytoscape 3.8.0 with ClueGO [33] for network analysis. Each node represents a biological function with a *p*-value < 0.05. Phosphosites identified in all stretching periods underwent cluster analysis by the bioconductor package “Mfuzz” [34]. We further applied DAVID [35] to annotate biological functions enriched in different clusters.

### 4.9. Transcriptomics Data Analysis

Transcriptomics data were downloaded from Gene Expression Omnibus (GEO) with accession number GSE15411 and GSE80161. We normalized and analyzed these transcriptomics data with the R package “limma” [78]. The significance of the transcriptomics data was defined as a *p*-value < 0.05. We performed Gene Set Enrichment Analysis (GSEA) [79] of differentially expressed genes. Enriched biological functions were further clustered using Reduce and Visualize Gene Ontology (REVIGO) [80].

### 4.10. Immunofluorescence Staining

Cells were seeded onto poly-L-lysine–coated flexible chambers and cultured for 1 d before undergoing cyclic stretching for 24 h or culturing under static conditions for 24 h. Before fixation, MitoTracker (Thermo Fisher Scientific, Waltham, MA, USA), a mitochondrial staining reagent, was diluted 10,000-fold in a serum-free medium and loaded into cell culture chambers for 30 min. Cells were then fixed in 4% formaldehyde (Sigma-Aldrich, St. Louis, MO, USA), permeabilized with 0.1% Triton X-100 (Sigma-Aldrich, St. Louis, MO, USA) for 15 min at room temperature, and then incubated with 1% BSA (BioShop, Burlington, ON, Canada) in PBS as a blocking buffer for 30 min at room temperature. Subsequently, cells were labeled with mouse monoclonal anti-actin antibody (Millipore, Billerica, MA, USA; 1:1000) and rabbit monoclonal anti-myosin IIa antibody (Cell Signaling Technology, Danvers, MA, USA 1:50) at 4 °C overnight. After washing with PBST (PBS containing 0.05% Tween-20, Sigma-Aldrich) three times, cells were labeled with the corresponding secondary anti-mouse IgG-Alexa 488 (Invitrogen, Carlsbad, CA, USA; 1:1000) and anti-rabbit FITC-IgG (Millipore, Burlington, ON, USA; 1:200) for 1 h in the dark at room temperature. The cells were then washed three times with PBST and mounted with ProLong Gold reagent with DAPI (Invitrogen, CA, USA). Images were acquired using a Zeiss LSM 780 Confocal Microscope (Technology Commons in College of Life Science, National Taiwan University) optimized for simultaneous fluorescent imaging.

### 4.11. Mitochondrial Image Analysis

For the calculation of the mitochondrial length, we analyzed the confocal images taken by Icy (http://icy.bioimageanalysis.org/) [43]. We overlaid the initial images with Zen, the image operating software from Zeiss, and input the processed images into Icy. We set the mitochondrial channel (Channel 1) for further operation. First, we adjusted the image contraction to enhance the weak signals. Next, the protocol was set for customized programming, and we extracted the selected channel from imaged mitochondria. Then, the images were blurred for the networked mitochondria with a Gaussian filter tool, and we displayed the images to check the results. We further loaded the filtered results to a k-means thresholder for automatic adjustment, and output [81] and displayed the processed images with a displayer. Finally, the processed images were loaded into a spot detector, and we selected the parameter of 100 specificity of 3-pixel detection. The results showed the perimeters, areas, and contour levels of every detected spot [82].

### 4.12. Statistical Analysis

The two-tailed *t-*test was used to analyze the stretching-induced mitochondrial length change in A549 and IMR-90 cells. Every stretching group was compared to the static group, respectively. The differences between the two groups were considered to be statistically significant when *p* < 0.05. The symbols *, **, and *** indicate *p* < 0.05, < 0.01, and < 0.005, respectively. The results of the mitochondrial length experiments are represented as a dot plot with the mean ± standard deviation.

## 5. Conclusions

In this study, we applied a quantitative phosphoproteomic technique to reveal the celltype-specific and common phosphosites that respond to cyclic stretching. Furthermore, we found that cell realignment is a strategy for resisting continual stretching and showed that mitochondrial lengths were enhanced by cell stretching. We propose that cyclic stretching-induced phospho-level changes provide a new perspective on dynamic stretching experiments.

## Supplementary Materials

Supplementary materials can be found at https://www.mdpi.com/1422-0067/21/11/4074/s1. **Figure S1.** Gene expression data analysis; **Table S1.** Research of cyclic stretching; **Table S2.** Significantly regulated phosphosites in IMR-90; **Table S3.** Significantly regulated phosphosites in the A549 cell line.
