# Supplementary material for: Quantitative Phosphoproteomics Reveals Cell Alignment and Mitochondrial Length Change under Cyclic Stretching in Lung Cells
Source: Int J Mol Sci. 2020 Jun 7;21(11):4074. doi: 10.3390/ijms21114074 (PMC7312583; doi:10.3390/ijms21114074)
Supplement: Supplementary file 1 [file ijms-21-04074-s001.zip › Supplementary/Supplementary.pdf]

International Journal of *Molecular Sciences*

MDPI

Supplementary

Quantitative Phosphoproteomics Reveals Cell Alignment and Mitochondrial Length Change under Cyclic Stretching in Lung Cells

Wei-Hsuan Wang [1], Chia-Lang Hsu [2,3], Hsuan-Cheng Huang [4,*] and Hsueh-Fen Juan [1,2,5,*]

[1] Genome and Systems Biology Degree Program, Academia Sinica and National Taiwan University, Taipei 10617, Taiwan; weihsuan15@gmail.com
[2] Department of Life Science, National Taiwan University, Taipei 10617, Taiwan; chialanghsu@ntuh.gov.tw
[3] Department of Medical Research, National Taiwan University Hospital, Taipei 10002, Taiwan
[4] Institute of Biomedical Informatics, National Yang-Ming University, Taipei 11230, Taiwan
[5] Graduate Institute of Biomedical Electronics and Bioinformatics, National Taiwan University, Taipei 10617, Taiwan

* Correspondence: yukijuan@ntu.edu.tw; Tel: +886-2-3366-4536; Fax: +886-2-23673374 (H.-F.J).
hsuancheng@ym.edu.tw; Tel: +886-2-28267357; Fax: +886-2-28202508; (H.-C.H.)

The Supplementary Information contains two sections:

I. Supplementary Figure

Figure S1. Gene expression data analysis.

II. Supplementary Tables (separate Microsoft Excel files)

Table S1. Researches of cyclic stretch

Table S2. Significantly regulated phosphosites in IMR-90

Table S3 Significantly regulated phosphosites in A549 cell line

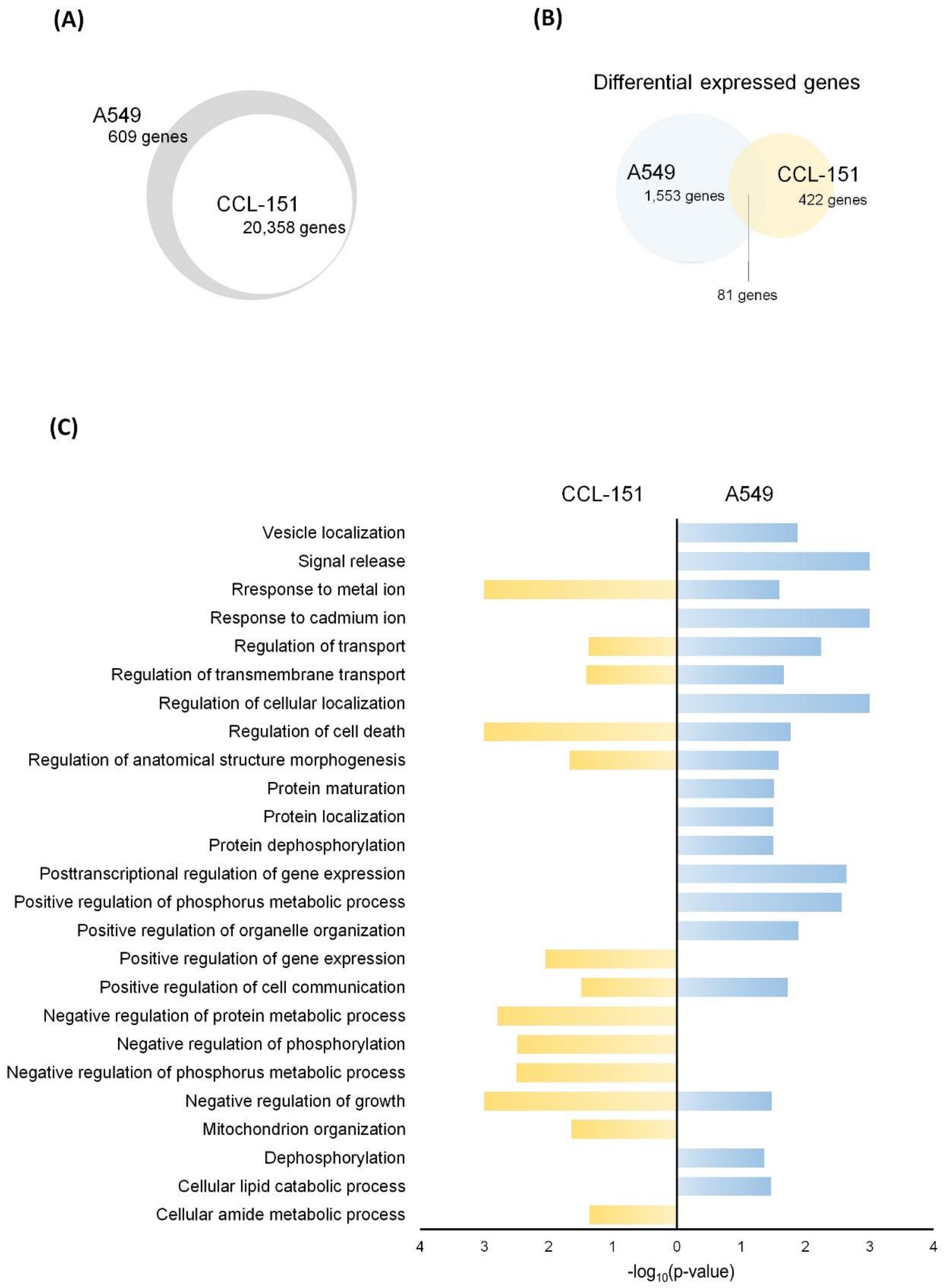

Figure S1. Gene expression data analysis. **(A).** All genes identified in the A549 and CCL-151 cell lines. **(B).** Venn diagram showing the overlap in the sets of differentially expressed genes. **(C).** Gene ontology analysis of differential genes in A549 and CCL-151 cell lines. Blue bar represents the enrich p value in A549 and yellow bar represents the p value in CCL-151.
